# Identifying and engineering the ideal microbial terpenoid production host

**DOI:** 10.1007/s00253-019-09892-y

**Published:** 2019-05-25

**Authors:** Sandra Moser, Harald Pichler

**Affiliations:** 1 Austrian Centre of Industrial Biotechnology (acib GmbH), Petersgasse 14, 8010 Graz, Austria; 2 Institute of Molecular Biotechnology, NAWI Graz, BioTechMed Graz, Graz University of Technology, Petersgasse 14/2, 8010 Graz, Austria

**Keywords:** Yeast, Bacteria, Terpenoids, Microbial production hosts, Cell engineering, Metabolic engineering

## Abstract

More than 70,000 different terpenoid structures are known so far; many of them offer highly interesting applications as pharmaceuticals, flavors and fragrances, or biofuels. Extraction of these compounds from their natural sources or chemical synthesis is—in many cases—technically challenging with low or moderate yields while wasting valuable resources. Microbial production of terpenoids offers a sustainable and environment-friendly alternative starting from simple carbon sources and, frequently, safeguards high product specificity. Here, we provide an overview on employing recombinant bacteria and yeasts for heterologous de novo production of terpenoids. Currently, *Escherichia coli* and *Saccharomyces cerevisiae* are the two best-established production hosts for terpenoids. An increasing number of studies have been successful in engineering alternative microorganisms for terpenoid biosynthesis, which we intend to highlight in this review. Moreover, we discuss the specific engineering challenges as well as recent advances for microbial production of different classes of terpenoids. Rationalizing the current stages of development for different terpenoid production hosts as well as future prospects shall provide a valuable decision basis for the selection and engineering of the cell factory(ies) for industrial production of terpenoid target molecules.

## Introduction

Terpenoids, i.e., terpenes and their functionalized derivatives, constitute one of the largest and structurally most diverse groups of natural compounds with over 70,000 different chemical structures (as listed in the *Dictionary of Natural Products* database (Vickers et al. 2017)). Although the majority of terpenoids have been found in plants, they also occur in insects (Laurent et al. 2003; Šobotník et al. 2010), in bacteria (Yamada et al. 2015), and in fungi (Quin et al. 2014). In accordance with their structural diversity, the functions of terpenoids range from mediating symbiotic or antagonistic interactions between organisms to electron transfer, protein prenylation, or contribution to membrane fluidity (Gershenzon and Dudareva 2007; Wriessnegger and Pichler 2013; Pichersky and Raguso 2016). These properties render terpenoids highly interesting for various applications, such as pharmaceuticals, flavors and fragrances, biofuels and fuel additives, or in agriculture as pesticides (Wang et al. 2005; Zwenger and Basu 2008; Bohlmann and Keeling 2008; George et al. 2015). Many of these compounds are still extracted from their natural sources, in most cases plants, although this approach often suffers from seasonal and geographical variations in supply and quality. For example, low yields or even lack of sufficient plant material was demonstrated in the case of the potent anticancer drug Taxol (paclitaxel) that had been found in the bark of mature pacific yew trees. It was calculated that 2–3 million pacific yew trees would have to be sacrificed per year to cover the demand for cancer treatment in the USA only (Suffness 1995).

**Fig. 1:**
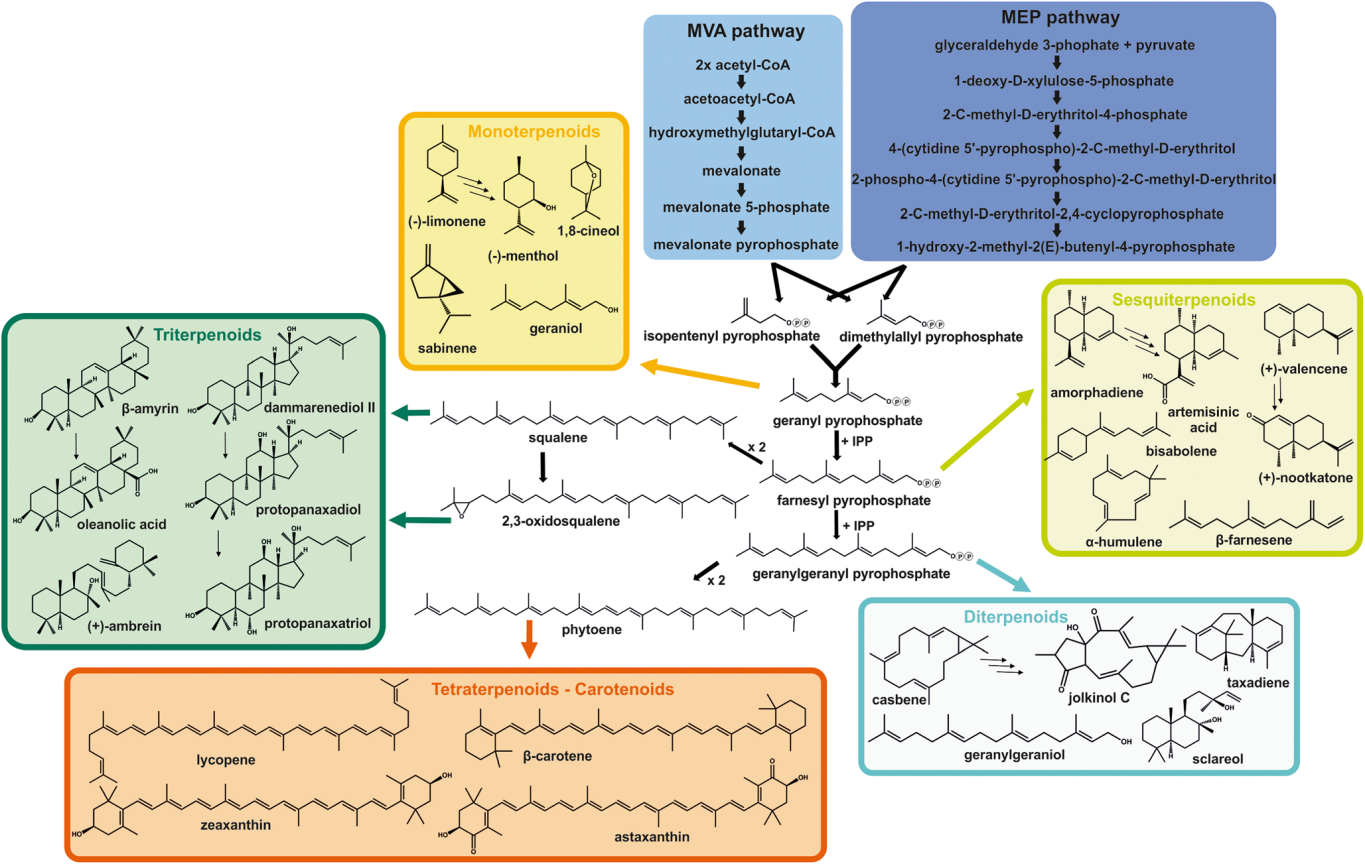
Overview of precursor production for terpenoid biosynthesis starting with the mevalonate (MVA) pathway (most eukaryotes) or the 2-*C*-methyl-D-erythritol-4-phosphate (MEP) pathway (bacteria and plant chloroplasts) and examples for different terpenoid classes derived from these prenyl diphosphate precursors

As an alternative supply route for many compounds, chemical synthesis has been established successfully (Jansen and Shenvi 2014). However, taking into account the progress made during the last two decades, biotechnological production of terpenoids now offers some major benefits. As recently analyzed for the example of C_13_-apocarotenoids (Cataldo et al. 2016), these advantages include renewable starting material, increased product specificity, mild process conditions, and the possibility to generate products considered natural. The latter feature is gaining importance especially in the fields of flavors and nutraceuticals. Microbial production hosts can start terpenoid biosynthesis from simple carbon sources due to endogenous metabolic pathways generating the universal precursors for all terpenoids, namely, isopentenyl diphosphate (IPP) and dimethylallyl diphosphate (DMAPP) (Fig. 1). The 2-*C*-methyl-D-erythritol-4-phosphate (MEP) pathway (also called DXP pathway) occurs in most bacteria as well as in plant chloroplasts and algae (Rohmer 1999) while the mevalonate (MVA) pathway is present in most eukaryotes, including plant cytosol, archaea, and eubacteria (Miziorko 2011). As shown in Fig. 1, condensation of two or more of the previously mentioned C_5_ molecules, IPP and DMAPP, leads to the formation of the larger prenyl diphosphate compounds farnesyl diphosphate (FPP), geranyl diphosphate (GPP), or geranylgeranyl diphosphate (GGPP) which represent the pool of precursors for terpenoid biosynthesis. Terpenoids are classified according to the number of carbon atoms they contain, starting from monoterpenoids (C_10_), sesquiterpenoids (C_15_), diterpenoids (C_20_) to triterpenoids (C_30_), and tetraterpenoids (carotenoids, C_40_) (Fig. 1). Additionally, a few special classes of terpenoids have been described such as hemiterpenoids (C_5_) (Li et al. 2018), sesterterpenoids (C_25_) (Wang et al. 2013a), sesquarterpenoids (C_35_) (Sato 2013), and polyterpenoids (> C_40_) (Swiezewska and Danikiewicz 2005) which will not be discussed in more detail within in this review.

## Metabolic engineering of microbial hosts for recombinant terpenoid production

The MEP and the MVA pathways constitute the two main targets of cell engineering approaches aiming to enhance terpenoid productivity. To increase precursor levels, one possibility is to focus on key enzymes in precursor supply that might be flux-limiting. Examples for such enzymes include, amongst others, DXP synthase (*dxs*) and isopentenyl diphosphate isomerase (*idi*) in the MEP pathway, which have been overexpressed in numerous studies of metabolic engineering of *Escherichia coli* (Albrecht et al. 1999; Kim and Keasling 2001; Leonard et al. 2010). Also in yeast, overexpression of either selected genes such as truncated HMG-CoA reductase 1 (t*HMG*) and FPP synthase (*ERG20*) (Ro et al. 2006; Shiba et al. 2007; Ignea et al. 2011) or even the whole MVA pathway (Westfall et al. 2012) significantly increased terpenoid yields. Another possibility to secure enhanced precursor supply is the expression of heterologous pathway genes. Martin et al. (2003) integrated the MVA pathway of *Saccharomyces cerevisiae* into *E. coli* in addition to the native MEP pathway, which greatly enhanced supply of prenyl diphosphate molecules. It is hypothesized that this effect can be attributed to the lack of tight regulation of the heterologous pathway by the host cell (Martin et al. 2003). Several subsequent studies demonstrated the positive impact of heterologous pathway gene expression in *E. coli* on precursor production (Tabata and Hashimoto 2004; Tsuruta et al. 2009; Yoon et al. 2009; Zhao et al. 2011). In contrast, results for the opposite approach, namely, introduction of the MEP pathway of *E. coli* into yeast, have been far from being as successful. This is especially relevant since, when starting from glucose, the MEP pathway in *E. coli* has a theoretically higher carbon efficiency due to the carbon loss of acetyl-CoA formation for the MVA pathway. Moreover, efficiency of both pathways is also highly dependent of the selected carbon source (Gruchattka et al. 2013). Only recently, a functional substitution of the native MVA pathway by a heterologous MEP pathway has been achieved in *S. cerevisiae*. The last two enzymes of the MEP pathway, IspG and IspH, which contain iron–sulfur clusters and also require additional redox partners, seem to be the major bottlenecks as they cannot easily be expressed in soluble fashion in yeast (Kirby et al. 2016).

Strikingly, expression of heterologous MVA pathway genes turned out to be beneficial for yeast hosts in some cases, especially when the upper part of the pathway was targeted (Hansen 2011; Li et al. 2013; Peng et al. 2017). Particularly in yeast(s), it is essential to downregulate the endogenous ergosterol biosynthesis pathway, which is competing for precursors. A certain level of ergosterol is essential for cell viability and proliferation, though (Daum et al. 1998). Very often, this has been achieved by exchanging native promoters for weaker ones that are responsive to glucose (Scalcinati et al. 2012) or methionine (Asadollahi et al. 2008) levels in the cultivation medium or to intracellular ergosterol levels (Yuan and Ching 2015). Another possibility that has been described recently was to tag the competing enzyme for degradation. This strategy has been successful both for reducing squalene synthase levels by fusing it to a C-terminal peptide recognized by the endoplasmic reticulum-associated protein degradation mechanism in a sesquiterpenoid-producing yeast (Peng et al. 2017). In a similar approach aiming to improve a monoterpenoid-producing strain by targeting FPP synthase for degradation (Peng et al. 2018), an N-terminal degron was added to the enzyme. To ensure efficient channeling of prenyl precursors to heterologous terpenoid biosynthesis, bringing precursor pathway enzymes in close proximity to terpene synthases has been the strategy in several examples in the literature. Both attempts of direct fusion of enzymes (Albertsen et al. 2011; Wang et al. 2011; Zhou et al. 2012; Baadhe et al. 2013; Yang et al. 2016) and employing assembly domains (Zhao et al. 2016) have been beneficial in both *E. coli* and various yeast species as well as for various classes of terpenoids.

Particularly in yeasts—with only one pathway providing prenyl diphosphate precursors for terpenoid biosynthesis—the issue of acetyl-CoA supply for the mevalonate pathway has been addressed (reviewed recently in more detail also by Vickers et al. (2017)). A pyruvate dehydrogenase (PDH) bypass was engineered that by providing additional acetyl-CoA—through overexpression of native acetaldehyde dehydrogenase(s) together with a *Salmonella enterica* acetyl-CoA synthetase variant—clearly increased flux through the mevalonate pathway resulting in a further twofold increase in amorphadiene levels in the best strain available (Shiba et al. 2007). Based on this strategy, acetyl-CoA supply for terpenoid production was pushed even further by engineering a push–pull block strategy that enhanced production of the sesquiterpene α-santalene fourfold. This increase in productivity was achieved by overexpressing a native alcohol dehydrogenase that converts ethanol to acetaldehyde and thereby channels it for additional acetyl-CoA supply (push). Furthermore, the first enzyme in the mevalonate pathway, acetyl-CoA C-acetyltransferase (pull), was overexpressed while reactions in the glyoxylate cycle competing for acetyl-CoA were inhibited (block) (Chen et al. 2013). A different approach, also aiming to improve overall acetyl-CoA supply, was to additionally utilize the mitochondrial acetyl-CoA pool by expressing the terpene synthase both in mitochondria and in the cytosol (Farhi et al. 2011). Engineering of central carbon metabolism for terpenoid biosynthesis was shifted to a new level by Meadows et al. (2016) who rendered *S. cerevisiae* more efficient in terms of ATP consumption and carbon flux. Endogenous pathways were replaced by heterologous metabolic reactions. For example, the previously described PDH bypass was substituted with an acetaldehyde dehydrogenase acylating (ADA) from *Dickeya zeae* which reduced the metabolic cost of farnesene by 18 ATPs, while expression of bacteria-derived xylulose-5-phosphate specific phosphoketolase and phosphotransacetylase circumvented CO_2_-emitting reactions. In addition, oxygen demand—of great importance for large-scale production—was decreased by astonishing 75%. Combined, these engineering approaches resulted in > 130 g L^−1^ of β-farnesene, by far the highest value reported so far for recombinant terpenoid production (Meadows et al. 2016).

For more detailed information on host engineering, we refer the reader to several excellent reviews that focus either on specific hosts, especially *E. coli* (Li and Wang 2016; Ward et al. 2018) and *S. cerevisiae* (Paramasivan and Mutturi 2017; Vickers et al. 2017; Zhang et al. 2017) or on strain engineering for selected targets such as isoprene (Ye et al. 2016) mono- (Zebec et al. 2016) or diterpenoids (Kemper et al. 2017), lycopene (Ma et al. 2016a), or fragrance and flavor molecules (Carroll et al. 2016).

## Alternative microbial hosts for terpenoid production

To date, the majority of studies that aimed for microbial terpenoid production were based on *E. coli* or *S. cerevisiae* as production chassis. Accordingly, the highest terpenoid titers have been reported for these two hosts (Tsuruta et al. 2009; Westfall et al. 2012; Meadows et al. 2016). This preference can mainly be attributed to the extensive knowledge of genomics, genetic engineering, metabolism, and cell biology of these two microbes, which was available already two decades ago when metabolic engineering for terpenoid production was still in its infancy. In addition, also fast growth and relatively simple cultivation conditions are properties of high importance when selecting a host for production at industrial scale. Therefore, especially for bulk chemical production for which the efficient utilization of each supplied carbon atom is of high economic importance, these two organisms still remain to be the first choice for production of most terpenoid molecules. However, considering recent developments of genetic engineering tools for other microorganisms (reviewed by Cho et al. (2018) and Raschmanová et al. (2018)), potential advantages of alternative production hosts over the two model organisms gain momentum. Although current terpenoid titers in alternative hosts may still lack economic feasibility, future metabolic engineering approaches will benefit from already established large-scale production processes of other valuable compounds by various microorganisms such as amino acids from *Corynebacterium glutamicum* (Ikeda and Takeno 2013) or heterologous proteins secreted from *Bacillus subtilis* (Schallmey et al. 2004). To achieve economic and sustainable production, the utilization of cheap, preferably nonsugar/nonfood carbon sources plays a major role. Substrates such as glycerol, ethanol, or methanol have been successfully employed for cultivation of *Pichia pastoris*, *Yarrowia lipolytica*, or *Methylobacterium extorquens* for terpenoid production (Matthäus et al. 2014; Wriessnegger et al. 2014; Sonntag et al. 2015; Czajka et al. 2018)*.* Meanwhile, even lignocellulosic feedstocks become more amenable, although for most microorganisms, substantial cell engineering is necessary to achieve sufficient yields on this nutrient source (Wei et al. 2015a; Wendisch et al. 2016; Niehus et al. 2018).

Autotrophic bacteria, such as the cyanobacteria *Synechococcus sp.* and *Synechocystis sp.*, *Rhodobacter sphaeroides*, or *Cupriavidus necator*, have been engineered for terpenoid production, in some cases actually utilizing CO_2_ as carbon source (Beekwilder et al. 2013; Choi et al. 2016; Formighieri and Melis 2017; Lee et al. 2017; Krieg et al. 2018). Photosynthetic bacteria are of specific interest as they are natural, high-level producers of terpenoids, more precisely pigments such as carotenoids, and therefore already operate the necessary metabolic pathways which can be further engineered to improve terpenoid yields (Pattanaik and Lindberg 2015; Su et al. 2018). Another factor that may influence the choice of production host is the type of enzyme(s) required to obtain the target molecule(s). While many terpene synthases can be expressed solubly in diverse hosts, expression of cytochrome P450 enzymes (CYP450s) which functionalize terpenes and, thereby, contribute to the great diversity of terpenoids has been challenging in many cases (Renault et al. 2014). Most CYP450s of plant origin are membrane-anchored to the endoplasmic reticulum (ER). Accordingly, functional expression of these enzymes in bacteria often is poor compared with eukaryotic hosts. Furthermore, CYP450s require coexpression of CYP450 reductases (CPRs) also inserted into the ER membrane as reviewed by Renault et al. (2014). Yet, functional CYP450/CPR coexpression in yeast(s) may bring along its issues as well, e.g., CPR instability, that may be cured by coexpression of *ICE2* (Emmerstorfer et al. 2015). Another issue that should be considered when selecting a microbial chassis and that we discuss in the next section in more detail is the toxicity of intermediates or terminal products on the microbial hosts themselves, which can considerably lower the yields. Therefore, genetically amenable bacteria that, by nature, are more tolerant to solvents, such as *Pseudomonas putida* or *B. subtilis* (Sardessai and Bhosle 2002; Nielsen et al. 2009), might be advantageous for terpenoid production. Ultimately, aiming for commercial applications, the selection of an alternative host might allow more freedom to operate since engineering of terpenoid biosynthesis in *E. coli* or *S. cerevisiae* is already restricted due to broad patent claims.

**Fig. 2:**
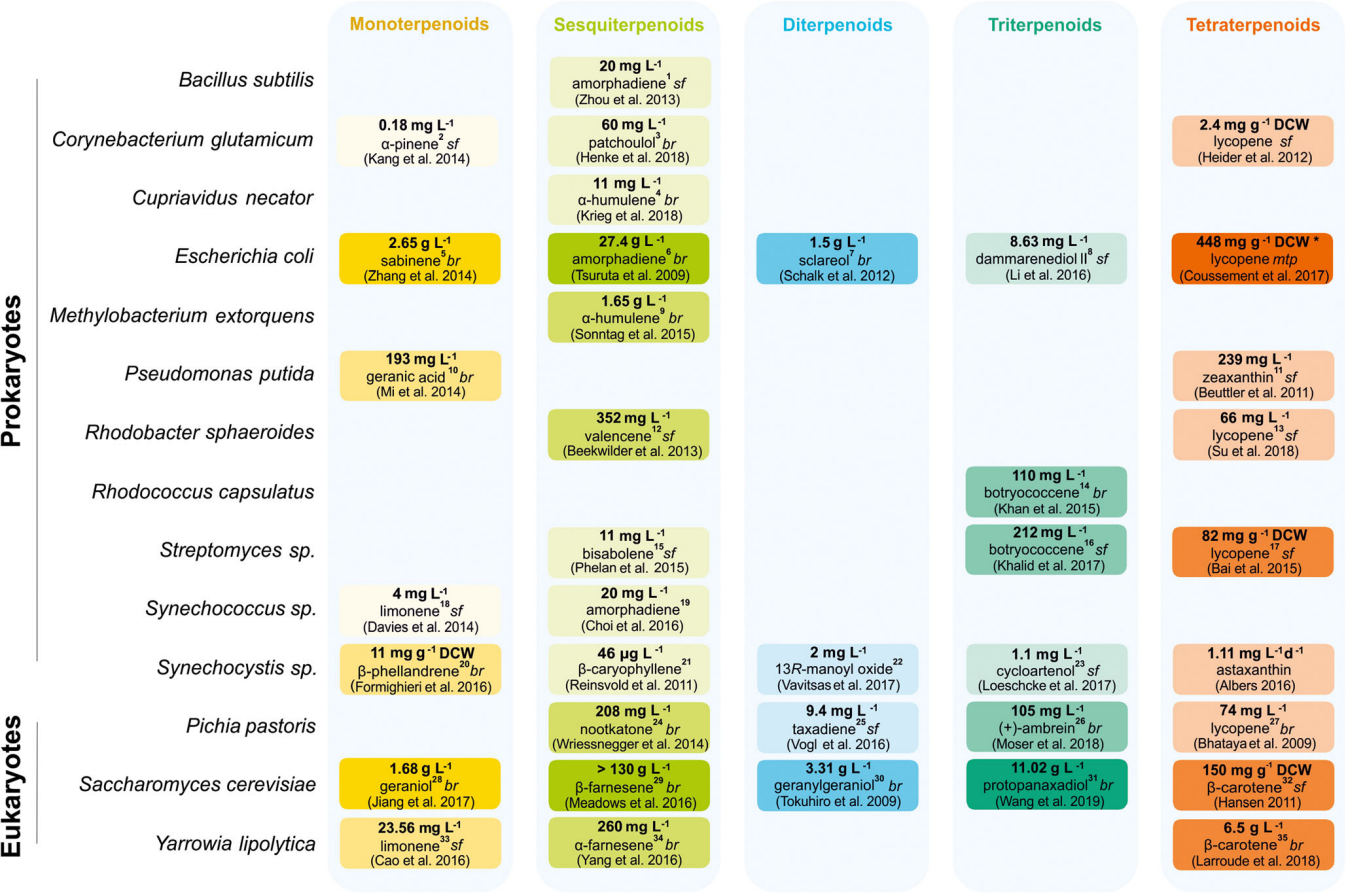
Highest reported value for each terpenoid class produced by different microbial hosts. Color intensity correlates to produced amounts for each class. Mode of cultivation: shake flask (sf), bioreactor (br), microtiter plate (mtp). If no cultivation mode is stated, details were not elaborated in literature, or the values could not be clearly assigned to one of the three modes of cultivation. Since titers and yields cannot easily be correlated due to considerable variations in growth and cell densities of different species and engineered strains, values were taken as stated in literature, preferably as titer (mg L^−1^ or g L^−1^), otherwise as space time yield (mg L^−1^ d^−1^) or specific yield (mg g^−1^ dry cell weight (DCW)). Superscript numbers behind the terpenoid molecule refer to footnotes with additional information on production time frame and eventually specific yield or space time yield, if available. Values that resulted from our own calculations based on available data in the respective study are given in italics. Asterisk indicates that this work has been substantially challenged by other authors (see Bian et al. 2018). ^1^Induction for 2 days; cultivation in 14 mL [sic!] Falcon tubes; ^2^induction for 52 h; specific yield 20.4 μg g^−1^ DCW; ^3^cultivation for 142 h; maximum space time yield 18 mg L^−1^d^−1^; specific yield 2 mg g^−1^ DCW; ^4^cultivation for 7 days; specific yield 17 mg g^−1^ DCW; maximum space time yield 0.08 mg L^−1^ h^−1^; ^5^12 h of growth and 24 h of induction; maximum specific productivity 0.018 g h^−1^ g^−1^ DCW; ^6^cultivation rounds lasted between 120 and 160 h; ^7^cultivation lasted close to 2 days including 20 h of induction; ^8^induction for 48 h; ^9^after 104 h of induction; average space time yield 14.6 mg L^−1^ h^−1^; specific yield 55 mg g^−1^ DCW; ^10^growth for 24 h and induction for 48 h; ^11^48 h of induction; ^12^cultivation for 72 h; ^13^cultivation for 168 h; specific yield 10.32 mg g^−1^ DCW; ^14^cultivation for 110 h; specific yield 16.7 mg g^−1^ DCW; ^15^cultivation for 72 h; ^16^cultivation for 9 days; ^17^cultivation for 5 days; ^18^cultivation for 96 h; highest rate 50 μg L^−1^ h^−1^; ^19^cultivation for 10 days; specific productivity over a 48 h culture 0.492 mg L^−1^OD_730_^−1^; ^20^cultivation for 48 h;^21^ cultivation for 1 week; specific yield 3.7 μg g^−1^ DCW; ^22^2 days of cultivation; specific yield 0.98 mg g^−1^ DCW; ^23^specific yield 2.06 mg g^−1^ DCW; specific titer 0.92 mg L^−1^OD^−1^; ^24^induction for 108 h; ^25^cultivation for 60 h; ^26^induction for 74 h; ^27^cultivation for 39.5 h; specific yield 4.6 mg g^−1^ DCW; ^28^after 130 h of cultivation; ^29^after 2 weeks; maximum space time yield 2.24 g L^−1^ h^−1^; ^30^206 h of cultivation; specific yield 70.9 mg g^−1^ DCW; ^31^144 h of fermentation; specific yield 76.9 mg g^−1^ DCW; ^32^cultivation for 48 h; ^33^cultivation for 3 days; specific yield 1.36 mg g^−1^ DCW; ^34^cultivation for 120 h; specific yield 33.98 mg g^−1^ DCW; ^35^cultivation for 122 h; specific yield 90 mg g^−1^ DCW

In the following section, we survey the specific challenges of microbial de novo biosynthesis for each terpenoid class in more detail. We provide an overview on how far various microbial hosts have been developed to reach industrially feasible terpenoid titers (see also Fig. 2).

## Microbial production of different terpenoid classes

### Monoterpenoids

The precursor molecule of monoterpenoids, geranyl diphosphate (GPP), is formed by condensation of dimethylallyl diphosphate (DMAPP) and isopentenyl diphosphate (IPP) (Fig. 1). Only low levels of endogenous GPP can be detected in microorganisms as most of GPP is efficiently converted to farnesyl pyrophosphate (FPP) by condensation with another molecule of IPP (Anderson et al. 1989; Thulasiram and Poulter 2006). In order to ensure sufficient precursor supply for monoterpenoid biosynthesis in microbial hosts, two different approaches have proven to be successful. Expression of heterologous GPP synthases from plant yielded improved monoterpenoid levels in *E. coli* (Yang et al. 2013; Alonso-Gutierrez et al. 2013; Zhang et al. 2014), *S. cerevisiae* (Ignea et al. 2011), *P. putida* (Mi et al. 2014), and *C. glutamicum* (Kang et al. 2014). Alternatively, the native FPP synthases of *E. coli* (Zhou et al. 2014) and *S. cerevisiae* (Fischer et al. 2011; Ignea et al. 2015) have been engineered to primarily yield GPP.

A major issue of microbial monoterpenoid production concerns the toxicity of these compounds to their production hosts. Apparent detrimental effects on membrane integrity have been described for different bacteria including *E. coli* as well as for *S. cerevisiae* (Sikkema et al. 1995; Trombetta et al. 2005). One possibility to overcome product toxicity is to perform biphasic cultivations for in situ extraction using dibutyl phthalate (Brennan et al. 2012), diisononyl phthalate (Willrodt et al. 2014), or dodecane (Alonso-Gutierrez et al. 2013) which also prevents loss of these highly volatile compounds. The same strategy is valid for cultures producing sesquiterpenoids. Another way is to heterologously express efflux pumps, a concept successfully implemented both in *E. coli* (Dunlop et al. 2011) and *S. cerevisiae* (Wang et al. 2013b)*.* Recently, it has been demonstrated for *S. cerevisiae* that the toxic effect of limonene can primarily be attributed to disturbing cell wall integrity (Brennan et al. 2013). Accordingly, expression of a truncated form of tricalbin 3, a protein with possible involvement in cell wall integrity regulation, was found to be highly beneficial for increasing *S. cerevisiae* tolerance against limonene, β-pinene, and myrcene (Brennan et al. 2015). In *E. coli*, expression of a mutated alkyl hydroperoxidase reduced the accumulation of the spontaneous oxidation product of limonene, limonene hydroxide, which seems to constitute the actually toxic compound for microorganisms in the presence of limonene (Chubukov et al. 2016).

Currently, *E. coli* or *S. cerevisiae* constitute the most productive monoterpenoid hosts, with the reported titers being in the low g L^−1^ range. Engineered *E. coli* yielded 2.65 g L^−1^ of the biofuel precursor sabinene (Zhang et al. 2014), 0.9 g L^−1^ of limonene (Willrodt et al. 2014), or 0.97 g L^−1^ of α-pinene (Yang et al. 2013). Both Yang et al. (2013) and Zhang et al. (2014) employed an *E. coli* strain expressing a hybrid MVA pathway from *Enterococcus faecalis* and *S. cerevisiae* which was shown to be clearly superior to the native MEP pathway. The highest reported titers for *S. cerevisiae* were 1.68 g L^−1^ of geraniol after screening nine different synthases, improving expression thereof and fusing it to FPP synthase (Jiang et al. 2017) and 1.1 g L^−1^ of cineol by overexpressing, amongst others, a more stable variant of the HMGR isoenzyme HMG2 (K6R) as well as a chaperone (HSP90) (Ignea et al. 2011). Although levels are still markedly lower, some alternative hosts exhibit a significantly higher resistance to monoterpenoids, rendering them interesting production chassis for this terpenoid class. In particular, *P. putida* offers high tolerance to monoterpenoids (Speelmans et al. 1998), and engineered strains have been successfully applied for de novo production of geranic acid yielding 193 mg L^−1^ (Mi et al. 2014) as well as for conversion of 1,8-cineole (Mi et al. 2016) and limonene (Mirata et al. 2009). The study by Mi et al. (2014) underlined the potential advantage of *P. putida* as monoterpenoid producer as it exhibited a markedly higher resistance—by at least a factor of 6—to geranic acid in comparison with both *S. cerevisiae* and *E. coli* (Mi et al. 2014)*.* Also, the oleaginous yeast *Y. lipolytica* was engineered to produce 23.6 mg L^−1^ of limonene (Cao et al. 2016) and 7 mg L^−1^ of linalool (Cao et al. 2017). Other hosts that had been engineered for monoterpenoid production include cyanobacteria for the production of limonene (Davies et al. 2014) or β-phellandrene (Formighieri and Melis 2016) with the titers being in the low mg L^−1^ range. For the production of α- and β-pinene in *C. glutamicum*, product levels are still in the low, triple-digit μg L^−1^ dimension (Kang et al. 2014).

In contrast to the above described biosynthesis of terpenoid backbones, further modifications of the hydrocarbons, as for example the conversion of limonene to menthol catalyzed by an enzyme cascade involving CYP450s from *Mentha spp.* (Turner and Croteau 2004), still remain a major challenge in microbial hosts. To our knowledge, no de novo biosynthesis of menthol from simple carbon source has been described, yet. However, a few approaches have been described that successfully produced menthol from pathway intermediates added externally (Toogood et al. 2015; Currin et al. 2018). Moreover, production and subsequent hydroxylation of limonene to another product, perillyl alcohol, in *E. coli* were reported to yield around ~ 100 mg L^−1^ of functionalized monoterpene (Alonso-Gutierrez et al. 2013).

### Sesquiterpenoids

To date, the most successful examples of microbial terpenoid production all fall into the class of sesquiterpenoids—which is not counterintuitive considering the essential nature of FPP-derived metabolites, e.g., sterols in eukaryotes. By far, the highest titers at > 130 g L^−1^ have been reported for production of β-farnesene—a building block for products ranging from cosmetics to fuel—using engineered *S. cerevisiae*. While synthesis of β-farnesene from FPP requires only a single enzyme, i.e., β-farnesene synthase, the major challenge was to modify carbon metabolism towards economic production of this bulk chemical by reducing ATP consumption and oxygen demand while improving carbon flux (Meadows et al. 2016), as discussed above. A far more complex biosynthetic route involving CYP450 activity and yielding in sufficient terpenoid for industrial scale was the production of precursors for artemisinin, an antimalarial drug. In *S. cerevisiae*, > 40 g L^−1^ of amorphadiene (Westfall et al. 2012) and 25 g L^−1^ of artemisinic acid (Paddon et al. 2013) that can be chemically converted to artemisinin were produced. In comparison with these values, the maximum titers reported for *E. coli* are 8.74 g L^−1^ of β-farnesene (You et al. 2017) and 27.4 g L^−1^ of amorphadiene (Tsuruta et al. 2009). Similarly, for two more sesquiterpenoids that can be produced in the low g L^−1^ range, *S. cerevisiae* appears to be superior to *E. coli* at the moment. Bisabolene levels reached 5.2 g L^−1^ after screening of a yeast deletion collection (Özaydın et al. 2013) while a principal component analysis of proteomics (PCAP) study for *E. coli* resulted in 1.15 g L^−1^ of bisabolene (Alonso-Gutierrez et al. 2015). An early patent of Millis et al. (2001) described a *S. cerevisiae* strain capable of producing 4.95 g L^−1^ of farnesol while another study on isoprenoid alcohol production in *E. coli* reported farnesol levels of 1.4 g L^−1^ (Zada et al. 2018). In contrast, engineering of *E. coli* for production of (−)-α-bisabolol and subsequent upscaling yielded 9.1 g L^−1^ which surpasses values reported for *S. cerevisiae* (Han et al. 2016).

Partially, based on the extensive work done in the two best-established production hosts, engineering of a few other microorganisms has advanced far enough to achieve sesquiterpenoid production in 3-digit mg L^−1^ range or higher. Sesquiterpenoid levels produced in such microbial hosts include for example 1.65 g L^−1^ of α-humulene in *M. extorquens*, which is remarkable considering that this bacterium was, until then, mainly known as a model organism for methylotrophy and not as a production chassis (Sonntag et al. 2015). Another methylotrophic microorganism, the yeast *P. pastoris*, was successfully employed for the functional expression of a CYP450/CPR pair in addition to valencene synthase, resulting in 208 mg L^−1^ of the grapefruit flavor (+)-nootkatone (Wriessnegger et al. 2014). The oleaginous yeast *Y. lipolytica* was engineered to produce 260 mg L^−1^ of α-farnesene by expressing a recombinant FPP synthase/α-farnesene synthase protein fusion in a strain modified for improved precursor production (Yang et al. 2016). Expression of a valencene synthase in a *R. sphaeroides* strain with a heterologous MVA pathway yielded 352 mg L^−1^ of valencene, a major aspect being the selection of a well-expressing synthase. In the same study, this synthase was also tested in a wild-type *S. cerevisiae* strain, but titers were considerably higher for the nonengineered bacterial host, thereby highlighting the potential of this phototrophic bacterium (Beekwilder et al. 2013). Also, a few other organisms were engineered for sesquiterpenoid biosynthesis such as *C. glutamicum* producing 60 mg L^−1^ of patchoulol (Henke et al. 2018), as well as 2.4 mg L^−1^ of valencene (Frohwitter et al. 2014) or *B. subtilis* yielding 20 mg L^−1^ of amorphadiene (Zhou et al. 2013). A β-caryophyllene synthase was introduced into the cyanobacterium *Synechocystis sp.* PCC6803 yielding 3.7 μg g^−1^ DCW (Reinsvold et al. 2011) while *Streptomyces venezuelae* was engineered to produce 10.5 mg L^−1^ of bisabolene (Phelan et al. 2015). In the latter study, also more complex carbon sources, such as cellobiose or ionic liquid-pretreated switchgrass, were successfully tested for bisabolene production, although titers were lower than with optimized medium. Further very interesting studies with regard to feedstock utilization were the production of 20 mg L^−1^ of amorphadiene (Choi et al. 2016) and 4.6 mg L^−1^ of α-farnesene (Lee et al. 2017) by *Synechococcus elongatus* PCC 7942, and remarkable 17 mg g^−1^ DCW of α-humulene by *C. necator* (Krieg et al. 2018), using CO_2_ as sole carbon source in all cases.

Beyond artemisinic acid and (+)-nootkatone, also some further examples of de novo biosynthesis and subsequent functionalization of sesquiterpenes catalyzed by CYP450s have been described, although titers are still relatively low. *E. coli* has been engineered to produce 105 mg L^−1^ of 8-hydroxycadinene (Chang et al. 2007) and—in a different study—an equivalent amount of costunolide which required heterologous expression of two CYP450s (Yin et al. 2015). *S. cerevisiae* was engineered to produce 50 mg L^−1^ of the dihydroxylated capsidiol (Takahashi et al. 2007) and, very recently, 40 mg L^−1^ of zerumbone which required biosynthesis of α-humulene, subsequent hydroxylation catalyzed by a CYP450, and conversion by a zerumbone synthase variant (Zhang et al. 2018b)*.*

Due to the extensive work done in the field of microbial sesquiterpenoid production within the last two decades, most of the terpenoids that are currently produced at commercial scale belong to this class. In addition to the already described high-level production of β-farnesene and artemisinic acid, also microbially produced flavor and fragrance molecules such as valencene or patchoulol are already on the market, as reviewed in more detail by Schempp et al. (2018).

### Diterpenoids

Biosynthesis of diterpenoids starts from GGPP which is formed by condensation of FPP with IPP (Fig. 1). GGPP levels in microbial production hosts are too low under standard conditions and, thus, need to be boosted for recombinant diterpenoid production. Different strategies have been employed; therefore, *S. cerevisiae* possesses a native GGPP synthase, *BTS1* (Jiang et al. 1995), that has been overexpressed either as single protein or as a part of fusion constructs with other pathway enzymes to enable efficient channeling of intermediates to push GGPP levels (Tokuhiro et al. 2009; Dai et al. 2012). Nevertheless, Bts1p catalytic activity is relatively low compared with GGPP synthases from other hosts (Ding et al. 2014). Therefore, in most studies engineering *E. coli* or *S. cerevisiae* for diterpenoid production, expression of heterologous GGPP synthases was the method of choice (Ajikumar et al. 2010; Morrone et al. 2010; Leonard et al. 2010; Dai et al. 2012; Schalk et al. 2012). Another possible solution was described by Ignea et al. (2015) who engineered the native FPP synthase of *S. cerevisiae* to a bifunctional enzyme that additionally produced significant amounts of GGPP. The benchmark for highest diterpenoid productivity is currently set by the production of 3.3 g L^−1^ (or 70.9 mg g^−1^ DCW) of geranylgeraniol which was achieved by creating fusion constructs of GGPP synthase with either FPP synthase or the endogenous diacylglycerol diphosphate phosphatase (Dpp1p) (Tokuhiro et al. 2009). A similar fusion approach was applied by Dai et al. (2012) for the generation of 488 mg L^−1^ of the tanshinone precursor miltiradiene, while Trikka et al. (2015) engineered *S. cerevisiae* for the production of 750 mg L^−1^ of the Ambrox precursor sclareol by knocking out six at first sight unrelated genes that had been identified in a carotenogenic screen. *E. coli* was employed to obtain 1.5 g L^−1^ of sclareol using two optimized synthases from Clary sage (Schalk et al. 2012). In the same host, 700 mg L^−1^ of levopimaradiene was reached through combinatorial mutagenesis of both GGPP and levopimaradiene synthase (Leonard et al. 2010).

The most publicity for microbial diterpenoid biosynthesis was attracted by the production of precursors of Taxol, an anticancer drug whose natural synthesis from GGPP involves 19 steps (Jennewein et al. 2004). Biosynthesis of taxadiene, the first intermediate in Taxol biosynthesis reached 1 g L^−1^ in *E. coli* (Ajikumar et al. 2010), while for *S. cerevisiae*, the highest reported value is 8.7 mg L^−1^ (Engels et al. 2008). Accordingly, CYP450-mediated generation of oxygenated taxanes, the next intermediates *en route* to Taxol, was reported to yield 570 mg L^−1^ in *E. coli* (Biggs et al. 2016). The same intermediates could only be produced by *S. cerevisiae* in a cocultivation strategy with taxadiene-producing *E. coli*, yet the levels were still clearly lower at 33 mg L^−1^ (Zhou et al. 2015). In the same study, another functionalized diterpenoid was produced in a similar way by combining miltiradiene-producing *E. coli* with *S. cerevisiae* expressing a CYP450 required for subsequent conversion to ferruginol at 18 mg L^−1^. Very interesting with regard to cheap feedstock utilization was the recent engineering of *E. coli* for the production of 364 mg L^−1^ of taxadiene utilizing corn steep liquor and glycerol as carbon source (Hirte et al. 2018). On the other hand, *S. cerevisiae* has been engineered to produce about 800 mg/L of jolkinol C and a record > 1 g L^−1^ of oxidized casbanes that are potential intermediates for the synthesis of various pharmaceuticals (Wong et al. 2018). To our knowledge, the only examples for diterpenoid production in alternative hosts are the biosynthesis of 360 μg g^−1^ DCW of geranyllinalool (Formighieri and Melis 2017) and 0.98 mg g^−1^ DCW of 13*R*-manoyl oxide (Vavitsas et al. 2017) in *Synechocystis* sp. PCC 6803 as well as the biosynthesis of 9.4 mg L^−1^ of taxadiene in *P. pastoris* (Vogl et al. 2016). Further analysis of the impact of these recombinant pathways and the resulting products on the cell as well as additional work on alleviating the bottleneck of GGPP supply will contribute to improving diterpenoid yields in alternative host.

### Triterpenoids

Condensation of two FPP molecules leads to formation of squalene which can either be used directly or get epoxidized to 2,3-oxidosqualene for subsequent steps of triterpenoid production. The majority of currently known triterpenoids found in higher organisms are formed from 2,3-oxidosqualene while prokaryotes usually take squalene as starting compound for triterpenoid formation (Abe et al. 1993) Thus, yeasts naturally producing both squalene and 2,3-oxidosqualene for ergosterol biosynthesis have a major starting advantage over *E. coli* and other prokaryotes that require expression of heterologous squalene- and 2,3-oxiosqualene synthases. Accordingly, the highest titers of triterpenoids have been reported for *S. cerevisiae.* Dai et al. (2013) engineered *S. cerevisiae* for production of 1.55 g L^−1^ of the ginsenoside precursor dammarenediol II, and upon coexpression of a CYP450/CPR pair, a remarkable amount of 1.19 g L^−1^ of protopanaxadiol was reported. Very recently, the production of dammarenediol II and subsequent conversion to protopanaxadiol was markedly improved through modular engineering of the MVA pathway combined with optimized CYP450 expression, finally yielding 11.02 g L^−1^ of protopanaxadiol (corresponding to 76.9 mg g^−1^ DCW) (Wang et al. 2019). Protopanaxadiol is converted to protopanaxatriol employing another CYP450 enzyme (Dai et al. 2014). Decoration of protopanaxadiol and protopanaxatriol through heterologous UDP-glycosyltransferases in *S. cerevisiae* yielded natural (Wang et al. 2015, 2019; Wei et al. 2015b) or novel (Liang et al. 2017) bioactive compounds*.* The highest titer reported for de novo production of a fully functionalized and glycosylated ginsenoside so far is 2.25 g L^−1^ of the potential anticancer drug Rh2 in *S. cerevisiae* (Wang et al. 2019). Dammarenediol II production has also been described in *E. coli*, requiring introduction of heterologous 2,3-oxidosqualene biosynthesis as well truncation of all N-terminal transmembrane domains of involved enzymes (Li et al. 2016) and *P. pastoris* (Zhao et al. 2016) but, in comparison with *S. cerevisiae*, at relatively low titers and specific yields of 8.63 mg L^−1^ and 1.04 mg g^−1^ DCW, respectively. α- and β-amyrin as well as their CYP450-derived products ursolic and oleanolic acid have been obtained in the low 3-digit mg L^−1^ range in *S. cerevisiae* (Lu et al. 2018). Recently, Zhao et al. (2018) enhanced oleanolic acid levels to 607 mg L^−1^ in *S. cerevisiae*. In addition to pushing precursor supply, the pairing of CYP450/CPR was optimized, and the galactose regulatory network was targeted to avoid negative effects on heterologous protein expression under the control of the ubiquitously used GAL promoter in the presence of glucose, which in addition also eliminated the requirement for cost-intensive supplementation with high amounts of galactose (Zhao et al. 2018). A very interesting approach with regard to overcoming the bottleneck of heterologous CYP450 expression in *S. cerevisiae* was described by Arendt et al. (2017) by engineering a yeast cell with significantly expanded endoplasmic reticulum to accommodate several, plant-derived CYP450 enzymes. This resulted in a 16-fold increase in production levels of medicagenic-28-*O*-glucoside, an oxidized and subsequently glycosylated derivative of β-amyrin (Arendt et al. 2017).

In contrast to *E. coli*, which is currently no competition for *S. cerevisiae* in triterpenoid production, the potential of several other hosts has been demonstrated in recent years. Our laboratory has recently reported the engineering of methylotrophic yeast *P. pastoris* for the biosynthesis of the squalene-derived (+)-ambrein, yielding 105 mg L^−1^ (Moser et al. 2018). Other hosts were modified to produce botryococcene in a similar range, such as *Streptomyces reveromyceticus* SN-593, a strain with a native mevalonate operon, that upon fine-tuning of expression of its global regulator of terpenoid biosynthesis, Fur22, yielded 212 mg L^−1^ (Khalid et al. 2017). *Rhodobacter capsulatus* produced 110 mg L^−1^ of botryococcene in an autotrophic cultivation setup supplying only CO_2_, H_2_, and O_2_ that yielded almost threefold more in titer compared with a glucose-based fed batch (Khan et al. 2015). The first synthesis of triterpenoids derived from 2,3-oxidosqualene in cyanobacteria was described by Loeschcke et al. (2017) who engineered *Synechocystis* sp. PCC 6803 for the production of cycloartenol, lupeol, and marneral. Additionally, traces of hydroxylated derivatives of lupeol and marneral were detected, presumably due to endogenous CYP450 activity.

### Tetraterpenoids (carotenoids)

Contrary to the terpenoid classes described above, for which most terpene synthases were derived from plants, carotenoid biosynthetic genes can also be found in many prokaryotes, fungi, or archaea (Sandmann 2002). This might constitute a possible advantage when heterologously overexpressing these biosynthetic genes in microbial production hosts. This hypothesis is supported by the fact that most studies in this section describing successful carotenoid production employed genes derived from microorganisms, fungi, or algae (Hansen 2011; Nam et al. 2013; Chen et al. 2016; Larroude et al. 2018). Upon condensation of two GGPP molecules, phytoene, the precursor for all carotenoids, is formed (Fig. 1). In noncarotenogenic hosts, this step requires heterologous expression of a phytoene synthase. In contrast to the other terpenoid classes, for which *E. coli* and *S. cerevisiae* are undisputedly the leading production hosts, the oleaginous yeast *Y. lipolytica* has been engineered to reach similar or even higher yields of different carotenoids. One major advantage of *Y. lipolytica* is its capability to form large lipid bodies in which high amounts of hydrophobic compounds, including carotenoids, can be stored (Matthäus et al. 2014). This ability can be further exploited upon strain engineering. For example, its native, already high acetyl-CoA flux can be engineered which renders *Y. lipolytica*, a highly promising host platform for terpenoid and lipid biosynthesis (Tai and Stephanopoulos 2013). Reported β-carotene yields are highest at 150 mg g^−1^ DCW for a *S. cerevisiae* strain that expressed a heterologous mevalonate pathway; each gene of which was selected from a different source, together with an ATP citrate lyase to push cytosolic acetyl-CoA levels (Hansen 2011). Remarkably, 90 mg g^−1^ DCW (corresponding to 6.5 mg L^−1^) was reported for *Y. lipolytica*, for which the ideal promoter–gene combinations were determined for all expression cassettes (Larroude et al. 2018), while *E. coli* produced 72.6 mg g^−1^ DCW after optimization of cultivation media composition. (Nam et al. 2013). Thus, these engineered hosts are competitive to natural microbial β-carotene producers currently used for production at industrial scale such as the microalga *Dunaliella salina* which has been reported to synthesize 37.3 mg g^−1^ DCW per day (García-González et al. 2005) or the fungus *Blakeslea trispora* which could be optimized to produce up to 55 mg g^−1^ DCW of β-carotene per day (Roukas et al. 2015). In contrast, yields of other hosts such as *C. glutamicum* or *P. pastoris* are relatively low in the μg g^−1^ DCW to single-digit mg g^−1^ DCW range (Araya-Garay et al. 2012; Henke et al. 2016).

For the β-carotene precursor lycopene, by far, the highest reported value was for an engineered *E. coli* strain that was reported to yield 448 mg g^−1^ DCW (Coussement et al. 2017). However, this report was challenged by Bian et al. (2018) very recently, claiming that due to missing and/or incomplete information, the earlier work could not be reproduced by others. For the yeasts *S. cerevisiae* (Chen et al. 2016) and *Y. lipolytica* (Schwartz et al. 2017), reported yields were at least one order of magnitude lower. Chen et al. (2016) produced 55.56 mg g^−1^ DCW of lycopene in a *S. cerevisiae* strain harboring several knockouts, including the YPL062W locus whose function was unclear at that time. Only very recently, it was determined by the same group that this locus functions as a promoter with major influence on terpenoid production and that a knockout positively influences production levels of all terpenoid classes (Chen et al. 2019). Respective lycopene production values for *C. glutamicum* or *P. pastoris* are markedly lower in the 1-digit mg g^−1^ DCW range (Bhataya et al. 2009; Heider et al. 2012). A quite high yield of 82 mg g^−1^ DCW was reported in a study analyzing regulatory elements in *Streptomyces avermitilis* that employed lycopene production as model pathway (Bai et al. 2015). In another study, the phototrophic and carotenogenic bacterium *R. sphaeroides* was further engineered to increase lycopene yields to 10 mg g^−1^ DCW (Su et al. 2018). Another carotenoid recombinantly produced in microorganisms is zeaxanthin, with *E. coli*, *P. putida*, or *C. glutamicum* all yielding in the mg g^−1^ DCW range (Beuttler et al. 2011; Heider et al. 2014; Shen et al. 2016). For astaxanthin, the yields reported for *C. glutamicum* (Henke et al. 2016), *E. coli* (Ma et al. 2016b), and *S. cerevisiae* (Zhou et al. 2017; Jin et al. 2018) are all in the low, 2-digit mg g^−1^ DCW range while production in *Synechocystis* PCC 6803 resulted in 1.11 mg L^−1^ d^−1^ (Albers 2016). This is still clearly lower than the values reported for microalgae, as for example the production of 77.2 mg g^−1^ DCW of astaxanthin in *Haematococcus pluvialis* (Kang et al. 2005).

On top of the very diverse applications of carotenoids themselves, also their cleavage products, the so-called apocarotenoids, are of high commercial value. Upon action of carotenoid cleavage dioxygenases (CCDs) at double bonds 9–10 and 9′–10′, β-ionone can be generated, which possesses interesting properties as fragrance and aroma compound. Remarkably, de novo biosynthesis of β-ionone from carotenoid-producing *E. coli* and *Y. lipolytica* has been yielding 500 and 380 mg L^−1^, respectively (Zhang et al. 2018a; Czajka et al. 2018). Slightly older work in *S. cerevisiae* had reported only around 5 mg L^−1^ (López et al. 2015). Furthermore, substantial amounts of α-ionone (480 mg L^−1^) can be produced in engineered *E. coli* strains upon CCD cleavage of ε-carotene (derived from lycopene) (Zhang et al. 2018a). Expression of a β-carotene 15,15′-oxygenase in engineered *E. coli* led to cleavage of ε-carotene, thereby generating 600 mg L^−1^ of the vitamin A alcohol retinal (Lee et al. 2012). Although the yields were clearly lower, also the saffron spice component crocetin could be produced de novo in both *E. coli* and *S. cerevisiae* through CCD-catalyzed cleavage of zeaxanthin and subsequent oxidation catalyzed by an aldehyde dehydrogenase (Chai et al. 2017; Giuliano et al. 2018).

## Conclusion and future perspective

Due to extensive, focused work on terpenoid production in *E. coli* and *S. cerevisiae* during the last two decades, these two microorganisms are the best-established hosts for a wide variety of different terpenoid compounds, in several cases already achieving industrially relevant yields (Westfall et al. 2012; Meadows et al. 2016; Larroude et al. 2018; Wang et al. 2019). However, quite a few studies on engineering of alternative hosts such as *Y. lipolytica*, *P. pastoris*, *M. extorquens*, or *S. avermitilis* for the production of selected terpenoid compounds demonstrated their potential, as yields are already competitive to the two “standard” hosts (Fig. 2). Especially for these novel hosts, it will be essential to analyze the impact of recombinant terpenoid production on host metabolism and accordingly adapt and balance pathway expression and regulation in order to optimize flux while avoiding feedback inhibition or toxicity by pathway intermediates. This approach will be facilitated by the wide and established range of tools for systems and synthetic biology that are available meanwhile. The positive impact of dynamic pathway regulation and metabolic balancing has been demonstrated in many recent studies for both *E. coli* and *S. cerevisiae* (Dahl et al. 2013; Xie et al. 2015; Meadows et al. 2016; Kim et al. 2016). Besides pathway engineering, also terpene synthases frequently play a major role as insufficient expression or activity can represent the major bottleneck. Selection of the best-performing enzyme from a library of potential synthases as well as further engineering towards enhanced selectivity and catalytic efficiency can greatly improve terpenoid yields (Leonard et al. 2010; Moses et al. 2014; Edgar et al. 2017; Abdallah et al. 2018). Furthermore, the productivity of one particular terpene synthase can differ markedly when employing different hosts as demonstrated by the comparative studies of Loeschcke et al. (2017) and Beekwilder et al. (2013). These works indicate the necessity for carefully adjusting the respective host for each product individually. In addition, also proteins that are not directly involved in the pathway itself or its regulation as well as cultivation conditions can have a major influence on host productivity. Such factors, whose positive impact on terpenoid production cannot always be rationally explained yet, were for example described by Trikka et al. (2015) for *S. cerevisiae*-producing sclareol and carotenoids or in our recent work for *P. pastoris* that has been engineered for the production of valencene, trans-nootkatol, and nootkatone (Wriessnegger et al. 2016). Mitigation of metabolic stress elicited through recombinant terpenoid production might be the reason for the described effects. Engineering of microbes for terpenoid production would be greatly facilitated by screening procedures for enhanced terpenoid synthesis as currently only a limited number of high-throughput methods are available for selected compounds (reviewed by Emmerstorfer-Augustin et al. (2016)).

## References

[CR1] Abdallah II, van Merkerk R, Klumpenaar E, Quax WJ (2018). Catalysis of amorpha-4,11-diene synthase unraveled and improved by mutability landscape guided engineering. Sci Rep.

[CR2] Abe I, Rohmer M, Prestwich GD (1993). Enzymatic cyclization of squalene and oxidosqualene to sterols and triterpenes. Chem Rev.

[CR3] Ajikumar PK, Xiao WH, Tyo KEJ, Wang Y, Simeon F, Leonard E, Mucha O, Phon TH, Pfeifer B, Stephanopoulos G (2010). Isoprenoid pathway optimization for Taxol precursor overproduction in *Escherichia coli*. Science (80).

[CR4] Albers SC (2016) Metabolic engineering of the cyanobacterium *Synechocystis sp.* PCC 6803 for the production of astaxanthin. Colorado State University. Libraries

[CR5] Albertsen L, Chen Y, Bach LS, Rattleff S, Maury J, Brix S, Nielsen J, Mortensen UH (2011). Diversion of flux toward sesquiterpene production in *Saccharomyces cerevisiae*. Appl Environ Microbiol.

[CR6] Albrecht M, Misawa N, Sandmann G (1999). Metabolic engineering of the terpenoid biosynthetic pathway of *Escherichia coli* for production of the carotenoids β-carotene and zeaxanthin. Biotechnol Lett.

[CR7] Alonso-Gutierrez J, Chan R, Batth TS, Adams PD, Keasling JD, Petzold CJ, Lee TS (2013). Metabolic engineering of *Escherichia coli* for limonene and perillyl alcohol production. Metab Eng.

[CR8] Alonso-Gutierrez J, Kim EM, Batth TS, Cho N, Hu Q, Chan LJG, Petzold CJ, Hillson NJ, Adams PD, Keasling JD, Garcia Martin H, Lee TS (2015). Principal component analysis of proteomics (PCAP) as a tool to direct metabolic engineering. Metab Eng.

[CR9] Anderson MS, Yarger JG, Burck CL, Poulter CD (1989). Farnesyl diphosphate synthetase. Molecular cloning, sequence, and expression of an essential gene from *Saccharomyces cerevisiae*. J Biol Chem.

[CR10] Araya-Garay JM, Ageitos JM, Vallejo JA, Veiga-Crespo P, Sánchez-Pérez A, Villa TG (2012). Construction of a novel *Pichia pastoris* strain for production of xanthophylls. AMB Express.

[CR11] Arendt P, Miettinen K, Pollier J, De Rycke R, Callewaert N, Goossens A (2017). An endoplasmic reticulum-engineered yeast platform for overproduction of triterpenoids. Metab Eng.

[CR12] Asadollahi MA, Maury J, Møller K, Nielsen KF, Schalk M, Clark A, Nielsen J (2008). Production of plant sesquiterpenes in *Saccharomyces cerevisiae*: effect of *ERG9* repression on sesquiterpene biosynthesis. Biotechnol Bioeng.

[CR13] Baadhe RR, Mekala NK, Parcha SR, Prameela Devi Y (2013). Combination of *ERG9* repression and enzyme fusion technology for improved production of amorphadiene in *Saccharomyces cerevisiae*. J Anal Methods Chem.

[CR14] Bai C, Zhang Y, Zhao X, Hu Y, Xiang S, Miao J, Lou C, Zhang L (2015). Exploiting a precise design of universal synthetic modular regulatory elements to unlock the microbial natural products in *Streptomyces*. Proc Natl Acad Sci.

[CR15] Beekwilder J, van Houwelingen A, Cankar K, van Dijk ADJ, de Jong RM, Stoopen G, Bouwmeester H, Achkar J, Sonke T, Bosch D (2013). Valencene synthase from the heartwood of Nootka cypress (*Callitropsis nootkatensis*) for biotechnological production of valencene. Plant Biotechnol J.

[CR16] Beuttler H, Hoffmann J, Jeske M, Hauer B, Schmid RD, Altenbuchner J, Urlacher VB (2011). Biosynthesis of zeaxanthin in recombinant *Pseudomonas putida*. Appl Microbiol Biotechnol.

[CR17] Bhataya A, Schmidt-Dannert C, Lee PC (2009). Metabolic engineering of *Pichia pastoris* X-33 for lycopene production. Process Biochem.

[CR18] Bian G, Ma T, Liu T (2018). *In vivo* platforms for terpenoid overproduction and the generation of chemical diversity. Methods Enzymol.

[CR19] Biggs BW, Lim CG, Sagliani K, Shankar S, Stephanopoulos G, De Mey M, Ajikumar PK (2016). Overcoming heterologous protein interdependency to optimize P450-mediated Taxol precursor synthesis in *Escherichia coli*. Proc Natl Acad Sci U S A.

[CR20] Bohlmann J, Keeling CI (2008). Terpenoid biomaterials. Plant J.

[CR21] Brennan TC, Turner CD, Krömer JO, Nielsen LK (2012) Alleviating monoterpene toxicity using a two-phase extractive fermentation for the bioproduction of jet fuel mixtures in Saccharomyces cerevisiae. Biotechnol Bioeng 109:2513–2522. 10.1002/bit.24536 10.1002/bit.24536 22539043

[CR22] Brennan TCR, Krömer JO, Nielsen LK (2013). Physiological and transcriptional responses of *Saccharomyces cerevisiae* to d-limonene show changes to the cell wall but not to the plasma membrane. Appl Environ Microbiol.

[CR23] Brennan TCR, Williams TC, Schulz BL, Palfreyman RW, Krömer JO, Nielsen LK (2015). Evolutionary engineering improves tolerance for replacement jet fuels in *Saccharomyces cerevisiae*. Appl Environ Microbiol.

[CR24] Cao X, Lv Y-B, Chen J, Imanaka T, Wei L-J, Hua Q (2016). Metabolic engineering of oleaginous yeast *Yarrowia lipolytica* for limonene overproduction. Biotechnol Biofuels.

[CR25] Cao X, Wei L-J, Lin J-Y, Hua Q (2017). Enhancing linalool production by engineering oleaginous yeast *Yarrowia lipolytica*. Bioresour Technol.

[CR26] Carroll AL, Desai SH, Atsumi S (2016). Microbial production of scent and flavor compounds. Curr Opin Biotechnol.

[CR27] Cataldo VF, López J, Cárcamo M, Agosin E (2016). Chemical vs. biotechnological synthesis of C13-apocarotenoids: current methods, applications and perspectives. Appl Microbiol Biotechnol.

[CR28] Chai F, Wang Y, Mei X, Yao M, Chen Y, Liu H, Xiao W, Yuan Y (2017). Heterologous biosynthesis and manipulation of crocetin in *Saccharomyces cerevisiae*. Microb Cell Factories.

[CR29] Chang MCY, Eachus RA, Trieu W, Ro D-K, Keasling JD (2007). Engineering *Escherichia coli* for production of functionalized terpenoids using plant P450s. Nat Chem Biol.

[CR30] Chen Y, Daviet L, Schalk M, Siewers V, Nielsen J (2013). Establishing a platform cell factory through engineering of yeast acetyl-CoA metabolism. Metab Eng.

[CR31] Chen Y, Xiao W, Wang Y, Liu H, Li X, Yuan Y (2016). Lycopene overproduction in *Saccharomyces cerevisiae* through combining pathway engineering with host engineering. Microb Cell Factories.

[CR32] Chen Y, Wang Y, Liu M, Qu J, Yao M, Li B, Ding M, Liu H, Xiao W, Yuan Y (2019). Primary and secondary metabolic effects of a key gene deletion (ΔYPL062W) in metabolically engineered terpenoid-producing *Saccharomyces cerevisiae*. Appl Environ Microbiol.

[CR33] Cho S, Shin J, Cho B-K, Cho S, Shin J, Cho B-K (2018). Applications of CRISPR/Cas system to bacterial metabolic engineering. Int J Mol Sci.

[CR34] Choi SY, Lee HJ, Choi J, Kim J, Sim SJ, Um Y, Kim Y, Lee TS, Keasling JD, Woo HM (2016). Photosynthetic conversion of CO_2_ to farnesyl diphosphate-derived phytochemicals (amorpha-4,11-diene and squalene) by engineered cyanobacteria. Biotechnol Biofuels.

[CR35] Chubukov V, Mukhopadhyay A, Petzold CJ, Keasling JD, Martín HG (2016). Synthetic and systems biology for microbial production of commodity chemicals. NPJ Syst Biol Appl.

[CR36] Coussement P, Bauwens D, Maertens J, De Mey M (2017). Direct combinatorial pathway optimization. ACS Synth Biol.

[CR37] Currin A, Dunstan MS, Johannissen LO, Hollywood KA, Vinaixa M, Jervis AJ, Swainston N, Rattray NJW, Gardiner JM, Kell DB, Takano E, Toogood HS, Scrutton NS (2018). Engineering the “missing link” in biosynthetic (−)-menthol production: bacterial isopulegone isomerase. ACS Catal.

[CR38] Czajka JJ, Nathenson JA, Benites VT, Baidoo EEK, Cheng Q, Wang Y, Tang YJ (2018). Engineering the oleaginous yeast *Yarrowia lipolytica* to produce the aroma compound β-ionone. Microb Cell Factories.

[CR39] Dahl RH, Zhang F, Alonso-Gutierrez J, Baidoo E, Batth TS, Redding-Johanson AM, Petzold CJ, Mukhopadhyay A, Lee TS, Adams PD, Keasling JD (2013). Engineering dynamic pathway regulation using stress-response promoters. Nat Biotechnol.

[CR40] Dai Z, Liu Y, Huang L, Zhang X (2012). Production of miltiradiene by metabolically engineered *Saccharomyces cerevisiae*. Biotechnol Bioeng.

[CR41] Dai Z, Liu Y, Zhang X, Shi M, Wang B, Wang D, Huang L, Zhang X (2013). Metabolic engineering of *Saccharomyces cerevisiae* for production of ginsenosides. Metab Eng.

[CR42] Dai Z, Wang B, Liu Y, Shi M, Wang D, Zhang X, Liu T, Huang L, Zhang X (2014). Producing aglycons of ginsenosides in bakers’ yeast. Sci Rep.

[CR43] Daum G, Lees ND, Bard M, Dickson R (1998). Biochemistry, cell biology and molecular biology of lipids of *Saccharomyces cerevisiae*. Yeast.

[CR44] Davies FK, Work VH, Beliaev AS, Posewitz MC (2014). Engineering limonene and bisabolene production in wild type and a glycogen-deficient mutant of *Synechococcus sp*. PCC 7002. Front Bioeng Biotechnol.

[CR45] Ding M, Yan H, Li L, Zhai F, Shang L, Yin Z, Yuan Y (2014). Biosynthesis of taxadiene in *Saccharomyces cerevisiae* : selection of geranylgeranyl diphosphate synthase directed by a computer-aided docking strategy. PLoS One.

[CR46] Dunlop MJ, Dossani ZY, Szmidt HL, Chu HC, Lee TS, Keasling JD, Hadi MZ, Mukhopadhyay A (2011). Engineering microbial biofuel tolerance and export using efflux pumps. Mol Syst Biol.

[CR47] Edgar S, Li F-S, Qiao K, Weng J-K, Stephanopoulos G (2017). Engineering of taxadiene synthase for improved selectivity and yield of a key Taxol biosynthetic intermediate. ACS Synth Biol.

[CR48] Emmerstorfer A, Wimmer-Teubenbacher M, Wriessnegger T, Leitner E, Müller, Kaluzna I, Schürmann M, Mink D, Zellnig G, Schwab H, Pichler H (2015) Over-expression of *ICE2* stabilizes cytochrome P450 reductase in *Saccharomyces cerevisiae* and *Pichia pastoris*. Biotechnol J 10:623–635. 10.1002/biot.201400780 10.1002/biot.201400780 25641738

[CR49] Emmerstorfer-Augustin A, Moser S, Pichler H (2016). Screening for improved isoprenoid biosynthesis in microorganisms. J Biotechnol.

[CR50] Engels B, Dahm P, Jennewein S (2008). Metabolic engineering of taxadiene biosynthesis in yeast as a first step towards Taxol (paclitaxel) production. Metab Eng.

[CR51] Farhi M, Marhevka E, Masci T, Marcos E, Eyal Y, Ovadis M, Abeliovich H, Vainstein A (2011). Harnessing yeast subcellular compartments for the production of plant terpenoids. Metab Eng.

[CR52] Fischer MJC, Meyer S, Claudel P, Bergdoll M, Karst F (2011). Metabolic engineering of monoterpene synthesis in yeast. Biotechnol Bioeng.

[CR53] Formighieri C, Melis A (2016). Sustainable heterologous production of terpene hydrocarbons in cyanobacteria. Photosynth Res.

[CR54] Formighieri C, Melis A (2017). Heterologous synthesis of geranyllinalool, a diterpenol plant product, in the cyanobacterium *Synechocystis*. Appl Microbiol Biotechnol.

[CR55] Frohwitter J, Heider SAE, Peters-Wendisch P, Beekwilder J, Wendisch VF (2014). Production of the sesquiterpene (+)-valencene by metabolically engineered *Corynebacterium glutamicum*. J Biotechnol.

[CR56] García-González M, Moreno J, Manzano JC, Florencio FJ, Guerrero MG (2005). Production of *Dunaliella salina* biomass rich in 9-cis-β-carotene and lutein in a closed tubular photobioreactor. J Biotechnol.

[CR57] George KW, Alonso-Gutierrez J, Keasling JD, Lee TS, Schrader J, Bohlmann J (2015). Isoprenoid drugs, biofuels, and chemicals-artemisinin, farnesene, and beyond. Advances in biochemical engineering/biotechnology.

[CR58] Gershenzon J, Dudareva N (2007). The function of terpene natural products in the natural world. Nat Chem Biol.

[CR59] Giuliano G, Ferrante P, Frusciante S, Diretto G, Pietrella M, Al-Babili S (2018) Carotenoid dioxygenase and methods for the biotechnological production in microorganisms and plants of compounds derived from saffron US9969989B2

[CR60] Gruchattka E, Hädicke O, Klamt S, Schütz V, Kayser O (2013). *In silico* profiling of *Escherichia coli* and *Saccharomyces cerevisiae* as terpenoid factories. Microb Cell Factories.

[CR61] Han GH, Kim SK, Yoon PK-S, Kang Y, Kim BS, Fu Y, Sung BH, Jung HC, Lee D-H, Kim S-W, Lee S-G (2016). Fermentative production and direct extraction of (−)-α-bisabolol in metabolically engineered *Escherichia coli*. Microb Cell Factories.

[CR62] Hansen J (2011) Method of producing isoprenoid compounds in yeast WO2011146833A1

[CR63] Heider SAE, Peters-Wendisch P, Wendisch VF (2012). Carotenoid biosynthesis and overproduction in *Corynebacterium glutamicum*. BMC Microbiol.

[CR64] Heider SAE, Wolf N, Hofemeier A, Peters-Wendisch P, Wendisch VF (2014). Optimization of the IPP precursor supply for the production of lycopene, decaprenoxanthin and astaxanthin by *Corynebacterium glutamicum*. Front Bioeng Biotechnol.

[CR65] Henke NA, Heider SAE, Peters-Wendisch P, Wendisch VF (2016). Production of the marine carotenoid astaxanthin by metabolically engineered *Corynebacterium glutamicum*. Mar Drugs.

[CR66] Henke NA, Wichmann J, Baier T, Frohwitter J, Lauersen KJ, Risse JM, Peters-Wendisch P, Kruse O, Wendisch VF (2018). Patchoulol production with metabolically engineered *Corynebacterium glutamicum*. Genes (Basel).

[CR67] Hirte M, Mischko W, Kemper K, Röhrer S, Huber C, Fuchs M, Eisenreich W, Minceva M, Brück TB (2018). From microbial upcycling to biology-oriented synthesis: combining whole-cell production and chemo-enzymatic functionalization for sustainable taxanoid delivery. Green Chem.

[CR68] Ignea C, Cvetkovic I, Loupassaki S, Kefalas P, Johnson CB, Kampranis SC, Makris AM (2011). Improving yeast strains using recyclable integration cassettes, for the production of plant terpenoids. Microb Cell Factories.

[CR69] Ignea C, Trikka FA, Nikolaidis AK, Georgantea P, Ioannou E, Loupassaki S, Kefalas P, Kanellis AK, Roussis V, Makris AM, Kampranis SC (2015). Efficient diterpene production in yeast by engineering Erg20p into a geranylgeranyl diphosphate synthase. Metab Eng.

[CR70] Ikeda M, Takeno S, Yukawa H, Inui M (2013). Amino acid production by *Corynebacterium glutamicum*. *Corynebacterium glutamicum*.

[CR71] Jansen DJ, Shenvi RA (2014). Synthesis of medicinally relevant terpenes: reducing the cost and time of drug discovery. Future Med Chem.

[CR72] Jennewein S, Wildung MR, Chau M, Walker K, Croteau R (2004). Random sequencing of an induced *Taxus* cell cDNA library for identification of clones involved in Taxol biosynthesis. Proc Natl Acad Sci U S A.

[CR73] Jiang Y, Proteau P, Poulter D, Ferro-Novick S (1995). BTS1 encodes a geranylgeranyl diphosphate synthase in *Saccharomyces cerevisiae*. J Biol Chem.

[CR74] Jiang GZ, Yao MD, Wang Y, Zhou L, Song TQ, Liu H, Xiao WH, Yuan YJ (2017). Manipulation of GES and ERG20 for geraniol overproduction in *Saccharomyces cerevisiae*. Metab Eng.

[CR75] Jin J, Wang Y, Yao M, Gu X, Li B, Liu H, Ding M, Xiao W, Yuan Y (2018). Astaxanthin overproduction in yeast by strain engineering and new gene target uncovering. Biotechnol Biofuels.

[CR76] Kang CD, Lee JS, Park TH, Sim SJ (2005). Comparison of heterotrophic and photoautotrophic induction on astaxanthin production by *Haematococcus pluvialis*. Appl Microbiol Biotechnol.

[CR77] Kang MK, Eom JH, Kim Y, Um Y, Woo HM (2014). Biosynthesis of pinene from glucose using metabolically-engineered *Corynebacterium glutamicum*. Biotechnol Lett.

[CR78] Kemper K, Hirte M, Reinbold M, Fuchs M, Brück T (2017). Opportunities and challenges for the sustainable production of structurally complex diterpenoids in recombinant microbial systems. Beilstein J Org Chem.

[CR79] Khalid A, Takagi H, Panthee S, Muroi M, Chappell J, Osada H, Takahashi S (2017). Development of a terpenoid-production platform in *Streptomyces reveromyceticus* SN-593. ACS Synth Biol.

[CR80] Khan NE, Nybo SE, Chappell J, Curtis WR (2015). Triterpene hydrocarbon production engineered into a metabolically versatile host—*Rhodobacter capsulatus*. Biotechnol Bioeng.

[CR81] Kim S-W, Keasling JD (2001). Metabolic engineering of the nonmevalonate isopentenyl diphosphate synthesis pathway in *Escherichia coli* enhances lycopene production. Biotechnol Bioeng.

[CR82] Kim SK, Han GH, Seong W, Kim H, Kim S-W, Lee D-H, Lee S-G (2016). CRISPR interference-guided balancing of a biosynthetic mevalonate pathway increases terpenoid production. Metab Eng.

[CR83] Kirby J, Dietzel KL, Wichmann G, Chan R, Antipov E, Moss N, Baidoo EEK, Jackson P, Gaucher SP, Gottlieb S, LaBarge J, Mahatdejkul T, Hawkins KM, Muley S, Newman JD, Liu P, Keasling JD, Zhao L (2016). Engineering a functional 1-deoxy-D-xylulose 5-phosphate (DXP) pathway in *Saccharomyces cerevisiae*. Metab Eng.

[CR84] Krieg T, Sydow A, Faust S, Huth I, Holtmann D (2018). CO_2_ to terpenes: autotrophic and electroautotrophic α-humulene production with *Cupriavidus necator*. Angew Chem Int Ed.

[CR85] Larroude M, Celinska E, Back A, Thomas S, Nicaud J-M, Ledesma-Amaro R (2018). A synthetic biology approach to transform *Yarrowia lipolytica* into a competitive biotechnological producer of β-carotene. Biotechnol Bioeng.

[CR86] Laurent P, Braekman J-C, Daloze D, Pasteels J (2003). Biosynthesis of defensive compounds from beetles and ants. Eur J Org Chem.

[CR87] Lee J-H, Choi J-G, Kim Y-S, Kim K-R, Kim S-W, Oh D-K (2012). Enhancement of retinal production by supplementing the surfactant Span 80 using metabolically engineered *Escherichia coli*. J Biosci Bioeng.

[CR88] Lee HJ, Lee J, Lee S-M, Um Y, Kim Y, Sim SJ, Choi J, Woo HM (2017). Direct conversion of CO_2_ to α-farnesene using metabolically engineered *Synechococcus elongatus* PCC 7942. J Agric Food Chem.

[CR89] Leonard E, Ajikumar PK, Thayer K, Xiao W-HW-H, Mo JD, Tidor B, Stephanopoulos G, Prather KLJ (2010). Combining metabolic and protein engineering of a terpenoid biosynthetic pathway for overproduction and selectivity control. Proc Natl Acad Sci.

[CR90] Li YF, Wang G (2016). Strategies of isoprenoids production in engineered bacteria. J Appl Microbiol.

[CR91] Li Q, Sun Z, Li J, Zhang Y (2013). Enhancing beta-carotene production in *Saccharomyces cerevisiae* by metabolic engineering. FEMS Microbiol Lett.

[CR92] Li D, Zhang Q, Zhou Z, Zhao F, Lu W (2016). Heterologous biosynthesis of triterpenoid dammarenediol-II in engineered *Escherichia coli*. Biotechnol Lett.

[CR93] Li M, Nian R, Xian M, Zhang H (2018). Metabolic engineering for the production of isoprene and isopentenol by *Escherichia coli*. Appl Microbiol Biotechnol.

[CR94] Liang H, Hu Z, Zhang T, Gong T, Chen J, Zhu P, Li Y, Yang J (2017). Production of a bioactive unnatural ginsenoside by metabolically engineered yeasts based on a new UDP-glycosyltransferase from *Bacillus subtilis*. Metab Eng.

[CR95] Loeschcke A, Dienst D, Wewer V, Hage-Hülsmann J, Dietsch M, Kranz-Finger S, Hüren V, Metzger S, Urlacher VB, Gigolashvili T, Kopriva S, Axmann IM, Drepper T, Jaeger K-E (2017). The photosynthetic bacteria *Rhodobacter capsulatus* and *Synechocystis sp.* PCC 6803 as new hosts for cyclic plant triterpene biosynthesis. PLoS One.

[CR96] López J, Essus K, Kim I, Pereira R, Herzog J, Siewers V, Nielsen J, Agosin E (2015). Production of β-ionone by combined expression of carotenogenic and plant CCD1 genes in *Saccharomyces cerevisiae*. Microb Cell Factories.

[CR97] Lu C, Zhang C, Zhao F, Li D, Lu W (2018). Biosynthesis of ursolic acid and oleanolic acid in *Saccharomyces cerevisiae*. AICHE J.

[CR98] Ma T, Deng Z, Liu T (2016). Microbial production strategies and applications of lycopene and other terpenoids. World J Microbiol Biotechnol.

[CR99] Ma T, Zhou Y, Li X, Zhu F, Cheng Y, Liu Y, Deng Z, Liu T (2016). Genome mining of astaxanthin biosynthetic genes from *Sphingomonas sp.* ATCC 55669 for heterologous overproduction in *Escherichia coli*. Biotechnol J.

[CR100] Martin VJJ, Pitera DJ, Withers ST, Newman JD, Keasling JD (2003). Engineering a mevalonate pathway in *Escherichia coli* for production of terpenoids. Nat Biotechnol.

[CR101] Matthäus F, Ketelhot M, Gatter M, Barth G (2014). Production of lycopene in the non-carotenoid-producing yeast *Yarrowia lipolytica*. Appl Environ Microbiol.

[CR102] Meadows AL, Hawkins KM, Tsegaye Y, Antipov E, Kim Y, Raetz L, Dahl RH, Tai A, Mahatdejkul-Meadows T, Xu L, Zhao L, Dasika MS, Murarka A, Lenihan J, Eng D, Leng JS, Liu C-L, Wenger JW, Jiang H, Chao L, Westfall P, Lai J, Ganesan S, Jackson P, Mans R, Platt D, Reeves CD, Saija PR, Wichmann G, Holmes VF, Benjamin K, Hill PW, Gardner TS, Tsong AE (2016). Rewriting yeast central carbon metabolism for industrial isoprenoid production. Nature.

[CR103] Mi J, Becher D, Lubuta P, Dany S, Tusch K, Schewe H, Buchhaupt M, Schrader J (2014). *De novo* production of the monoterpenoid geranic acid by metabolically engineered *Pseudomonas putida*. Microb Cell Factories.

[CR104] Mi J, Schewe H, Buchhaupt M, Holtmann D, Schrader J (2016). Efficient hydroxylation of 1,8-cineole with monoterpenoid-resistant recombinant *Pseudomonas putida* GS1. World J Microbiol Biotechnol.

[CR105] Millis J, Maurina-Brunker J, McMullin T (2001) Production of farnesol and geranylgeraniol US6689593B2

[CR106] Mirata MA, Heerd D, Schrader J (2009). Integrated bioprocess for the oxidation of limonene to perillic acid with *Pseudomonas putida* DSM 12264. Process Biochem.

[CR107] Miziorko HM (2011). Enzymes of the mevalonate pathway of isoprenoid biosynthesis. Arch Biochem Biophys.

[CR108] Morrone D, Lowry L, Determan MK, Hershey DM, Xu M, Peters RJ (2010). Increasing diterpene yield with a modular metabolic engineering system in *E. coli*: comparison of MEV and MEP isoprenoid precursor pathway engineering. Appl Microbiol Biotechnol.

[CR109] Moser S, Strohmeier GA, Leitner E, Plocek TJ, Vanhessche K, Pichler H (2018). Whole-cell (+)-ambrein production in the yeast *Pichia pastoris*. Metab Eng Commun.

[CR110] Moses T, Thevelein JM, Goossens A, Pollier J (2014). Comparative analysis of CYP93E proteins for improved microbial synthesis of plant triterpenoids. Phytochemistry.

[CR111] Nam H-K, Choi J-G, Lee J-H, Kim S-W, Oh D-K (2013). Increase in the production of β-carotene in recombinant *Escherichia coli* cultured in a chemically defined medium supplemented with amino acids. Biotechnol Lett.

[CR112] Niehus X, Crutz-Le Coq A-M, Sandoval G, Nicaud J-M, Ledesma-Amaro R (2018). Engineering *Yarrowia lipolytica* to enhance lipid production from lignocellulosic materials. Biotechnol Biofuels.

[CR113] Nielsen DR, Leonard E, Yoon S-H, Tseng H-C, Yuan C, Prather KLJ (2009). Engineering alternative butanol production platforms in heterologous bacteria. Metab Eng.

[CR114] Özaydın B, Burd H, Lee TS, Keasling JD (2013). Carotenoid-based phenotypic screen of the yeast deletion collection reveals new genes with roles in isoprenoid production. Metab Eng.

[CR115] Paddon CJ, Westfall PJ, Pitera DJ, Benjamin K, Fisher K, McPhee D, Leavell MD, Tai A, Main A, Eng D, Polichuk DR, Teoh KH, Reed DW, Treynor T, Lenihan J, Fleck M, Bajad S, Dang G, Dengrove D, Diola D, Dorin G, Ellens KW, Fickes S, Galazzo J, Gaucher SP, Geistlinger T, Henry R, Hepp M, Horning T, Iqbal T, Jiang H, Kizer L, Lieu B, Melis D, Moss N, Regentin R, Secrest S, Tsuruta H, Vazquez R, Westblade LF, Xu L, Yu M, Zhang Y, Zhao L, Lievense J, Covello PS, Keasling JD, Reiling KK, Renninger NS, Newman JD (2013). High-level semi-synthetic production of the potent antimalarial artemisinin. Nature.

[CR116] Paramasivan K, Mutturi S (2017). Progress in terpene synthesis strategies through engineering of *Saccharomyces cerevisiae*. Crit Rev Biotechnol.

[CR117] Pattanaik B, Lindberg P (2015). Terpenoids and their biosynthesis in cyanobacteria. Life (Basel, Switzerland).

[CR118] Peng B, Plan MR, Chrysanthopoulos P, Hodson MP, Nielsen LK, Vickers CE (2017). A squalene synthase protein degradation method for improved sesquiterpene production in *Saccharomyces cerevisiae*. Metab Eng.

[CR119] Peng B, Nielsen LK, Kampranis SC, Vickers CE (2018). Engineered protein degradation of farnesyl pyrophosphate synthase is an effective regulatory mechanism to increase monoterpene production in *Saccharomyces cerevisiae*. Metab Eng.

[CR120] Phelan RM, Sekurova ON, Keasling JD, Zotchev SB (2015). Engineering terpene biosynthesis in *Streptomyces* for production of the advanced biofuel precursor bisabolene. ACS Synth Biol.

[CR121] Pichersky E, Raguso RA (2016). Why do plants produce so many terpenoid compounds?. New Phytol.

[CR122] Quin MB, Flynn CM, Schmidt-Dannert C (2014). Traversing the fungal terpenome. Nat Prod Rep.

[CR123] Raschmanová H, Weninger A, Glieder A, Kovar K, Vogl T (2018). Implementing CRISPR-Cas technologies in conventional and non-conventional yeasts: current state and future prospects. Biotechnol Adv.

[CR124] Reinsvold RE, Jinkerson RE, Radakovits R, Posewitz MC, Basu C (2011). The production of the sesquiterpene β-caryophyllene in a transgenic strain of the cyanobacterium *Synechocystis*. J Plant Physiol.

[CR125] Renault H, Bassard J-E, Hamberger B, Werck-Reichhart D (2014). Cytochrome P450-mediated metabolic engineering: current progress and future challenges. Curr Opin Plant Biol.

[CR126] Ro D-K, Paradise EM, Ouellet M, Fisher KJ, Newman KL, Ndungu JM, Ho KA, Eachus RA, Ham TS, Kirby J, Chang MCY, Withers ST, Shiba Y, Sarpong R, Keasling JD (2006). Production of the antimalarial drug precursor artemisinic acid in engineered yeast. Nature.

[CR127] Rohmer M (1999). The discovery of a mevalonate-independent pathway for isoprenoid biosynthesis in bacteria, algae and higher plants. Nat Prod Rep.

[CR128] Roukas T, Varzakakou M, Kotzekidou P (2015). From cheese whey to carotenes by *Blakeslea trispora* in a bubble column reactor. Appl Biochem Biotechnol.

[CR129] Sandmann G (2002). Molecular evolution of carotenoid biosynthesis from bacteria to plants. Physiol Plant.

[CR130] Sardessai Y, Bhosle S (2002). Tolerance of bacteria to organic solvents. Res Microbiol.

[CR131] Sato T (2013). Unique biosynthesis of sesquarterpenes (C35 terpenes). Biosci Biotechnol Biochem.

[CR132] Scalcinati G, Knuf C, Partow S, Chen Y, Maury J, Schalk M, Daviet L, Nielsen J, Siewers V (2012). Dynamic control of gene expression in *Saccharomyces cerevisiae* engineered for the production of plant sesquitepene α-santalene in a fed-batch mode. Metab Eng.

[CR133] Schalk M, Pastore L, Mirata MA, Khim S, Schouwey M, Deguerry F, Pineda V, Rocci L, Daviet L (2012). Toward a biosynthetic route to sclareol and amber odorants. J Am Chem Soc.

[CR134] Schallmey M, Singh A, Ward OP (2004). Developments in the use of *Bacillus* species for industrial production. Can J Microbiol.

[CR135] Schempp FM, Drummond L, Buchhaupt M, Schrader J (2018). Microbial cell factories for the production of terpenoid flavor and fragrance compounds. J Agric Food Chem.

[CR136] Schwartz C, Frogue K, Misa J, Wheeldon I (2017). Host and pathway engineering for enhanced lycopene biosynthesis in *Yarrowia lipolytica*. Front Microbiol.

[CR137] Shen H-J, Cheng B-Y, Zhang Y-M, Tang L, Li Z, Bu Y-F, Li X-R, Tian G-Q, Liu J-Z (2016). Dynamic control of the mevalonate pathway expression for improved zeaxanthin production in *Escherichia coli* and comparative proteome analysis. Metab Eng.

[CR138] Shiba Y, Paradise EM, Kirby J, Ro D-K, Keasling JD (2007). Engineering of the pyruvate dehydrogenase bypass in *Saccharomyces cerevisiae* for high-level production of isoprenoids. Metab Eng.

[CR139] Sikkema J, de Bont JA, Poolman B (1995). Mechanisms of membrane toxicity of hydrocarbons. Microbiol Mol Biol Rev.

[CR140] Šobotník J, Jirošová A, Hanus R (2010). Chemical warfare in termites. J Insect Physiol.

[CR141] Sonntag F, Kroner C, Lubuta P, Peyraud R, Horst A, Buchhaupt M, Schrader J (2015). Engineering *Methylobacterium extorquens* for *de novo* synthesis of the sesquiterpenoid α-humulene from methanol. Metab Eng.

[CR142] Speelmans G, Bijlsma A, Eggink G (1998). Limonene bioconversion to high concentrations of a single and stable product, perillic acid, by a solvent-resistant *Pseudomonas putida* strain. Appl Microbiol Biotechnol.

[CR143] Su A, Chi S, Li Y, Tan S, Qiang S, Chen Z, Meng Y (2018). Metabolic redesign of *Rhodobacter sphaeroides* for lycopene production. J Agric Food Chem.

[CR144] Suffness M (1995). Taxol : science and applications.

[CR145] Swiezewska E, Danikiewicz W (2005). Polyisoprenoids: structure, biosynthesis and function. Prog Lipid Res.

[CR146] Tabata K, Hashimoto S-I (2004). Production of mevalonate by a metabolically-engineered *Escherichia coli*. Biotechnol Lett.

[CR147] Tai M, Stephanopoulos G (2013). Engineering the push and pull of lipid biosynthesis in oleaginous yeast *Yarrowia lipolytica* for biofuel production. Metab Eng.

[CR148] Takahashi S, Yeo Y, Greenhagen BT, McMullin T, Song L, Maurina-Brunker J, Rosson R, Noel JP, Chappell J (2007). Metabolic engineering of sesquiterpene metabolism in yeast. Biotechnol Bioeng.

[CR149] Thulasiram HV, Poulter CD (2006). Farnesyl diphosphate synthase: the art of compromise between substrate selectivity and stereoselectivity. J Am Chem Soc.

[CR150] Tokuhiro K, Muramatsu M, Ohto C, Kawaguchi T, Obata S, Muramoto N, Hirai M, Takahashi H, Kondo A, Sakuradani E, Shimizu S (2009). Overproduction of geranylgeraniol by metabolically engineered *Saccharomyces cerevisiae*. Appl Environ Microbiol.

[CR151] Toogood HS, Cheallaigh AN, Tait S, Mansell DJ, Jervis A, Lygidakis A, Humphreys L, Takano E, Gardiner JM, Scrutton NS (2015). Enzymatic menthol production: one-pot approach using engineered *Escherichia coli*. ACS Synth Biol.

[CR152] Trikka FA, Nikolaidis A, Athanasakoglou A, Andreadelli A, Ignea C, Kotta K, Argiriou A, Kampranis SC, Makris AM (2015). Iterative carotenogenic screens identify combinations of yeast gene deletions that enhance sclareol production. Microb Cell Factories.

[CR153] Trombetta D, Castelli F, Sarpietro MG, Venuti V, Cristani M, Daniele C, Saija A, Mazzanti G, Bisignano G (2005). Mechanisms of antibacterial action of three monoterpenes. Antimicrob Agents Chemother.

[CR154] Tsuruta H, Paddon CJ, Eng D, Lenihan JR, Horning T, Anthony LC, Regentin R, Keasling JD, Renninger NS, Newman JD (2009). High-level production of amorpha-4, 11-diene, a precursor of the antimalarial agent artemisinin, in *Escherichia coli*. PLoS One.

[CR155] Turner GW, Croteau R (2004). Organization of monoterpene biosynthesis in *Mentha*. Immunocytochemical localizations of geranyl diphosphate synthase, limonene-6-hydroxylase, isopiperitenol dehydrogenase, and pulegone reductase. Plant Physiol.

[CR156] Vavitsas K, Rue EØ, Stefánsdóttir LK, Gnanasekaran T, Blennow A, Crocoll C, Gudmundsson S, Jensen PE (2017). Responses of *Synechocystis sp*. PCC 6803 to heterologous biosynthetic pathways. Microb Cell Factories.

[CR157] Vickers CE, Williams TC, Peng B, Cherry J (2017). Recent advances in synthetic biology for engineering isoprenoid production in yeast. Curr Opin Chem Biol.

[CR158] Vogl T, Glieder A, Ajikumar PK (2016) Production of terpenes and terpenoids US20180094286A1

[CR159] Wang G, Tang W, Bidigare RR, Zhang L, Demain A (2005). Terpenoids as therapeutic drugs and pharmaceutical agents. Natural products.

[CR160] Wang C, Yoon S-H, Jang H-J, Chung Y-R, Kim J-Y, Choi E-S, Kim S-W (2011). Metabolic engineering of *Escherichia coli* for α-farnesene production. Metab Eng.

[CR161] Wang L, Yang B, Lin X-P, Zhou X-F, Liu Y (2013). Sesterterpenoids. Nat Prod Rep.

[CR162] Wang Y, Lim L, DiGuistini S, Robertson G, Bohlmann J, Breuil C (2013). A specialized ABC efflux transporter GcABC-G1 confers monoterpene resistance to *Grosmannia clavigera* , a bark beetle-associated fungal pathogen of pine trees. New Phytol.

[CR163] Wang P, Wei Y, Fan Y, Liu Q, Wei W, Yang C, Zhang L, Zhao G, Yue J, Yan X, Zhou Z (2015). Production of bioactive ginsenosides Rh2 and Rg3 by metabolically engineered yeasts. Metab Eng.

[CR164] Wang P, Wei W, Ye W, Li X, Zhao W, Yang C, Li C, Yan X, Zhou Z (2019). Synthesizing ginsenoside Rh2 in *Saccharomyces cerevisiae* cell factory at high-efficiency. Cell Discov.

[CR165] Ward VCA, Chatzivasileiou AO, Stephanopoulos G (2018). Metabolic engineering of *Escherichia coli* for the production of isoprenoids. FEMS Microbiol Lett.

[CR166] Wei N, Oh EJ, Million G, Cate JHD, Jin Y-S (2015). Simultaneous utilization of cellobiose, xylose, and acetic acid from lignocellulosic biomass for biofuel production by an engineered yeast platform. ACS Synth Biol.

[CR167] Wei W, Wang P, Wei Y, Liu Q, Yang C, Zhao G, Yue J, Yan X, Zhou Z (2015). Characterization of *Panax ginseng* UDP-glycosyltransferases catalyzing protopanaxatriol and biosyntheses of bioactive ginsenosides F1 and Rh1 in metabolically engineered yeasts. Mol Plant.

[CR168] Wendisch VF, Brito LF, Gil Lopez M, Hennig G, Pfeifenschneider J, Sgobba E, Veldmann KH (2016). The flexible feedstock concept in industrial biotechnology: metabolic engineering of *Escherichia coli*, *Corynebacterium glutamicum*, *Pseudomonas*, *Bacillus* and yeast strains for access to alternative carbon sources. J Biotechnol.

[CR169] Westfall PJ, Pitera DJ, Lenihan JR, Eng D, Woolard FX, Regentin R, Horning T, Tsuruta H, Melis DJ, Owens A, Fickes S, Diola D, Benjamin KR, Keasling JD, Leavell MD, McPhee DJ, Renninger NS, Newman JD, Paddon CJ (2012). Production of amorphadiene in yeast, and its conversion to dihydroartemisinic acid, precursor to the antimalarial agent artemisinin. Proc Natl Acad Sci U S A.

[CR170] Willrodt C, David C, Cornelissen S, Bühler B, Julsing MK, Schmid A (2014). Engineering the productivity of recombinant *Escherichia coli* for limonene formation from glycerol in minimal media. Biotechnol J.

[CR171] Wong J, de Rond T, D’Espaux L, van der Horst C, Dev I, Rios-Solis L, Kirby J, Scheller H, Keasling J (2018). High-titer production of lathyrane diterpenoids from sugar by engineered *Saccharomyces cerevisiae*. Metab Eng.

[CR172] Wriessnegger T, Pichler H (2013). Yeast metabolic engineering—targeting sterol metabolism and terpenoid formation. Prog Lipid Res.

[CR173] Wriessnegger T, Augustin P, Engleder M, Leitner E, Müller M, Kaluzna I, Schürmann M, Mink D, Zellnig G, Schwab H, Pichler H (2014). Production of the sesquiterpenoid (+)-nootkatone by metabolic engineering of *Pichia pastoris*. Metab Eng.

[CR174] Wriessnegger T, Moser S, Emmerstorfer-Augustin A, Leitner E, Müller M, Kaluzna I, Schürmann M, Mink D, Pichler H (2016). Enhancing cytochrome P450-mediated conversions in *P. pastoris* through RAD52 over-expression and optimizing the cultivation conditions. Fungal Genet Biol.

[CR175] Xie W, Ye L, Lv X, Xu H, Yu H (2015). Sequential control of biosynthetic pathways for balanced utilization of metabolic intermediates in *Saccharomyces cerevisiae*. Metab Eng.

[CR176] Yamada Y, Kuzuyama T, Komatsu M, Shin-Ya K, Omura S, Cane DE, Ikeda H (2015). Terpene synthases are widely distributed in bacteria. Proc Natl Acad Sci U S A.

[CR177] Yang J, Nie Q, Ren M, Feng H, Jiang X, Zheng Y, Liu M, Zhang H, Xian M (2013). Metabolic engineering of *Escherichia coli* for the biosynthesis of alpha-pinene. Biotechnol Biofuels.

[CR178] Yang X, Nambou K, Wei L, Hua Q (2016). Heterologous production of α-farnesene in metabolically engineered strains of *Yarrowia lipolytica*. Bioresour Technol.

[CR179] Ye L, Lv X, Yu H (2016). Engineering microbes for isoprene production. Metab Eng.

[CR180] Yin H, Zhuang Y, Li E, Bi H, Zhou W, Liu T (2015). Heterologous biosynthesis of costunolide in *Escherichia coli* and yield improvement. Biotechnol Lett.

[CR181] Yoon S-H, Lee S-H, Das A, Ryu H-K, Jang H-J, Kim J-Y, Oh D-K, Keasling JD, Kim S-W (2009). Combinatorial expression of bacterial whole mevalonate pathway for the production of β-carotene in *E. coli*. J Biotechnol.

[CR182] You S, Yin Q, Zhang J, Zhang C, Qi W, Gao L, Tao Z, Su R, He Z (2017). Utilization of biodiesel by-product as substrate for high-production of β-farnesene via relatively balanced mevalonate pathway in *Escherichia coli*. Bioresour Technol.

[CR183] Yuan J, Ching C-B (2015). Dynamic control of ERG9 expression for improved amorpha-4,11-diene production in *Saccharomyces cerevisiae*. Microb Cell Factories.

[CR184] Zada B, Wang C, Park J-B, Jeong S-H, Park J-E, Singh HB, Kim S-W (2018). Metabolic engineering of *Escherichia coli* for production of mixed isoprenoid alcohols and their derivatives. Biotechnol Biofuels.

[CR185] Zebec Z, Wilkes J, Jervis AJ, Scrutton NS, Takano E, Breitling R (2016). Towards synthesis of monoterpenes and derivatives using synthetic biology. Curr Opin Chem Biol.

[CR186] Zhang H, Liu Q, Cao Y, Feng X, Zheng Y, Zou H, Liu H, Yang J, Xian M (2014). Microbial production of sabinene—a new terpene-based precursor of advanced biofuel. Microb Cell Factories.

[CR187] Zhang Y, Nielsen J, Liu Z (2017). Engineering yeast metabolism for production of terpenoids for use as perfume ingredients, pharmaceuticals and biofuels. FEMS Yeast Res.

[CR188] Zhang C, Chen X, Lindley ND, Too H-P (2018). A “plug-n-play” modular metabolic system for the production of apocarotenoids. Biotechnol Bioeng.

[CR189] Zhang C, Liu J, Zhao F, Lu C, Zhao G-R, Lu W (2018). Production of sesquiterpenoid zerumbone from metabolic engineered *Saccharomyces cerevisiae*. Metab Eng.

[CR190] Zhao Y, Yang J, Qin B, Li Y, Sun Y, Su S, Xian M (2011). Biosynthesis of isoprene in *Escherichia coli* via methylerythritol phosphate (MEP) pathway. Appl Microbiol Biotechnol.

[CR191] Zhao C, Gao X, Liu X, Wang Y, Yang S, Wang F, Ren Y (2016). Enhancing biosynthesis of a ginsenoside precursor by self-assembly of two key enzymes in *Pichia pastoris*. J Agric Food Chem.

[CR192] Zhao Y, Fan J, Wang C, Feng X, Li C (2018). Enhancing oleanolic acid production in engineered *Saccharomyces cerevisiae*. Bioresour Technol.

[CR193] Zhou YJ, Gao W, Rong Q, Jin G, Chu H, Liu W, Yang W, Zhu Z, Li G, Zhu G, Huang L, Zhao ZK (2012). Modular pathway engineering of diterpenoid synthases and the mevalonic acid pathway for miltiradiene production. J Am Chem Soc.

[CR194] Zhou K, Zou R, Zhang C, Stephanopoulos G, Too H-P (2013). Optimization of amorphadiene synthesis in *Bacillus subtilis* via transcriptional, translational, and media modulation. Biotechnol Bioeng.

[CR195] Zhou J, Wang C, Yoon S-H, Jang H-J, Choi E-S, Kim S-W (2014). Engineering *Escherichia coli* for selective geraniol production with minimized endogenous dehydrogenation. J Biotechnol.

[CR196] Zhou K, Qiao K, Edgar S, Stephanopoulos G (2015). Distributing a metabolic pathway among a microbial consortium enhances production of natural products. Nat Biotechnol.

[CR197] Zhou P, Xie W, Li A, Wang F, Yao Z, Bian Q, Zhu Y, Yu H, Ye L (2017). Alleviation of metabolic bottleneck by combinatorial engineering enhanced astaxanthin synthesis in *Saccharomyces cerevisiae*. Enzym Microb Technol.

[CR198] Zwenger S, Basu C (2008). Plant terpenoids: applications and future potentials. Biotechnol Mol Biol Rev.

